# Clk/STY (cdc2-Like Kinase 1) and Akt Regulate Alternative Splicing and Adipogenesis in 3T3-L1 Pre-Adipocytes

**DOI:** 10.1371/journal.pone.0053268

**Published:** 2013-01-04

**Authors:** Pengfei Li, Gay Carter, Jacqueline Romero, Kathryn M. Gower, James Watson, Niketa A. Patel, Denise R. Cooper

**Affiliations:** 1 Department of Molecular Medicine, Morsani College of Medicine, University of South Florida, Tampa, Florida, United States of America; 2 Research Service, J.A. Haley Veterans Hospital, Tampa, Florida, United States of America; National Institute of Health - National Cancer Institute, United States of America

## Abstract

The development of adipocytes from their progenitor cells requires the action of growth factors signaling to transcription factors to induce the expression of adipogenic proteins leading to the accumulation of lipid droplets, induction of glucose transport, and secretion of adipokines signaling metabolic events throughout the body. Murine 3T3-L1 pre-adipocytes sequentially express all the proteins necessary to become mature adipocytes throughout an 8–10 day process initiated by a cocktail of hormones. We examined the role of Clk/STY or Clk1, a cdc2-like kinase, in adipogenesis since it is known to be regulated by Akt, a pivotal kinase in development. Inhibition of Clk1 by a specific inhibitor, TG003, blocked alternative splicing of PKCβII and expression of PPARγ1 and PPARγ2. SiRNA depletion of Clk1 resulted in early expression of PKCβII and sustained PKCβI expression. Since Clk1 is a preferred Akt substrate, required for phosphorylation of splicing factors, mutation of Clk1 Akt phosphorylation sites was undertaken. Akt sites on Clk1 are in the serine/arginine-rich domain and not the kinase domain. Mutation of single and multiple sites resulted in dysregulation of PKCβII, PKCβI, and PPARγ1&2 expression. Additionally, adipogenesis was blocked as assessed by Oil Red O staining, adiponectin, and Glut1 and 4 expression. Immunofluorescence microscopy revealed that Clk1 triple mutant cDNA, transfected into pre-adipocytes, resulted in excluding SRp40 (SFSR6) from co-localizing to the nucleus with PFS, a perispeckle specific protein. This study demonstrates the role of Akt and Clk1 kinases in the early differentiation of 3T3-L1 cells to adipocytes.

## Introduction

The development of mature adipocytes, or adipogenesis, is one of the most intensely studied models of cellular differentiation [1]. The development of obesity requires the continuous differentiation of new adipocytes throughout life and is implicated in insulin resistance, type 2 diabetes, hypertension and atherosclerosis, which make up the metabolic syndrome [2]. The best *in vitro* model for studying adipogenesis has been the 3T3-L1 pre-adipocyte [3], [4]. Transplantation of these cells into athymic mice results in the formation of a normal fat pad [5]. 3T3-L1 cells isolated by Green and colleague [3], [4], [5] commenced a large number of studies describing the properties of clonal preadipocytes that, when treated with appropriate agents, differentiate into mature fat cells after a 4–6 day period. These findings rose questions as to what cues stimulate adipogenesis.

The expression of transcription factors, peroxisome proliferator-activated receptor γ (PPAR γ) and CCAAT/enhancer binding protein α (CEBP α) are largely responsible for a permanent period of growth arrest followed by a differentiated phenotype [6]. This is only the beginning of differentiation, however, as pre-adipocytes further undergo changes in gene expression that promotes their final phenotype including proteins required for lipid storage, triacylglycerol lipolysis and release of free fatty acids, and glucose transport. Many of these events are regulated by adiponectin, an adipocyte secreted hormone with insulin-sensitizing, anti-inflammatory and anti-apoptotic functions [7], [8].

Although transcriptional control of adipogenesis is well documented, it does not describe the entire process. Messenger RNA splicing is a synonymous or post-transcriptional modification of pre-mRNA of eukaryotic cells, whereby noncoding introns are removed and exons are joined to provide new protein templates. Alternative splicing (AS) is a variation where exons of pre-mRNAs are linked by AS by inclusion or exclusion to produce transcripts with different protein coding sequences. It is predicted that >92–94% of genes undergo AS with variation between tissue types and developmental stages [9]. The process is also regulated by hormones as in the case of insulin regulating the splicing of protein kinase C (PKC) β II [10], [11], [12], [13], [14]. The signaling process regulating insulin action in splicing is the PI3Kinase/Akt/Clk1 kinase pathway [15]. Activation of Clk1, a cdc2-like kinase, by Akt allows for phosphorylation of targeted serine/arginine-rich (SR) splicing factors by Clk1 so that SR proteins can act as enhancers or repressors of splicing activity. PKC β ’s role in fat development is poorly understood. Mice deficient in PKCβ (I+II) are lean and resistant to diet induced obesity [16]. PKCβII phosphorylates Akt on Serine473 in mature 3T3-L1 adipocytes [17].

We demonstrated that PKC βII was spliced in 3T3-L1 adipocytes in a developmentally regulated manner [17]. 3T3-L1 pre-adipocytes express PKCβI at days 0 to 4 of differentiation *in vitro*. Between days 4–6 of the differentiation process, PKCβI splicing is suppressed while PKCβII splicing increases. We hypothesized that the PI3Kinase/Akt/Clk1 signaling pathway was important for adipoctye differentiation for regulating PKCβII splicing since pAkt(S473) is activated very early in adipogenesis. The role of Clk1 in this process was unknown. To examine this question, a chemical inhibitor of Clk1 and 4 isoforms, TG003, siRNA for Clk1, and mutants of Clk1 with Akt phosphorylation sites mutated to alanine were used to determine their effect on PKCβII splicing and the progression of adipogenesis by markers PPARγ adiponectin, GLUT4, Glut1, and Oil Red O staining of accumulating lipid droplets.

## Methods

### Cell Culture

Mouse 3T3-L1 pre-adipocytes obtained from American Type Tissue Culture repository, ATCC (Manassas, VA) were maintained and passaged as pre-confluent cultures in DMEM high glucose (Invitrogen, Carlsbad, CA) with 10% newborn calf serum (Sigma-Aldrich, St. Louis, MO) at 37°C and 10% CO_2_. Confluent cells were differentiated (day 0) in DMEM high glucose with 10% fetal bovine serum (Atlas Biological, Fort Collins, CO), 10 µg/mL bovine insulin (Sigma), 1 mM dexamethasone (Sigma), and 0.5 mM isobutyl-1-methylxanthine (Sigma). On day 2, media was replaced with DMEM high glucose, 10% FBS, and bovine insulin. Day 4 and afterwards, cells were cultured in DMEM high glucose plus 10% FBS.

### Mutation of Akt Sites in Clk1

Generation of Clk1 mutant cDNA was generated using QuickChange Site-Directed Mutagenesis Kit (Stratagene). The Clk1 template was denatured at 95°C. The mutagenic primers containing the desired mutation(s) were annealed at 55°C and primers extended using PfuUltra DNA polymerase at 68°C. The parental DNA was digested with Dpn I enzyme. The pure mutated DNA was transformed into competent cells and harvested. The mutated fragment was confirmed by sequencing.

### Transfection of Pre-adipocytes with siRNA and myc-Clk

Clk siRNA oligonucleotides were obtained from Dharmacon (SMARTpool). The DharmaFECT1 transfection reagent was used in siRNA transfections. 3T3-L1 cells were seeded in 6-well plates (80% confluent). SiRNA reagent was mixed with Opti-MEMI media (Invitrogen) and DharmaFECT1 reagent. The transfection mixture was added to each well afterwards. Non-target control siRNA (Dharmacon) was used as negative controls. The knock-down efficiency was measured at 24, 48 and 72 hour time points with RT-PCR. Myc-Clk over-expression was obtained using Amaxa Cell Line Nucleofector Kit L (Lonza). 3T3-L1 cells were trypsinized and suspended at 2×10^6^ cells/100 ul, then combined with 2 ug DNA, 100 ul cell line nucleofector solution L, selected nucleofector program A-033 to transfer, immediately transfer the sample into collagen pre-coated 6-well plate.

### Western Blot

3T3-L1 cells were harvested in lysis buffer containing protease inhibitors (Sigma*Fast* Protease Inhibitor Tablet, Sigma) and phosphatase inhibitors (Phosphatase Inhibitor Cocktail 1, Sigma). Lysates (40 µg) were run on an SDS-PAGE gel and transferred to Hybond-C Extra nitrocellulose membranes (Amersham, Piscataway, NJ). Membranes were blocked and probed in 5% non-fat dried milk. The only exception was when probing for PKCβII, where pig gelatin (Bio-Rad) was used for blocking (3%), primary (1%) and secondary (1%). Detection was performed using SuperSignal West Pico Chemiluminescent substrate (Pierce Biotechnology, Rockford, IL). Antibodies used were as follows: PPARγ 81B8, pIRβ Tyr 1150/1151, pAkt Ser473 #4058, and adiponectin #2789 (Cell Signaling, Boston, MA), GLUT-1, GLUT4 C-Terminus 07-1404 (Millipore, Billerica, MA), β-actin A5441 (Sigma). PKCβII NH2–(GC) EGFSFVNSEFLKPEVKS-COOH (aa 657–673) was raised by BioSynthesis Inc. (Lewisville, TX) and purified using Nab Protein A Plus Spin Kit (Pierce #89948). Monoclonal antibody 104 was from ATCC. SRp40 (SFSR5), SRp55 (SFSR6) antibodies were described previously [15].

### RT PCR

RNA was extracted using RNA Bee (Tel Test Inc., Friendswood, TX), according to manufacturer’s protocol. Reverse Transcriptase was performed using Omniscript RT kit (Qiagen, Valencia, CA, #205113) according to manufacturer’s protocol. PCR reactions were performed to determine Clk 1 mRNA levels using JumpStart Ready-Start amplification mix (Sigma) and previously published primers. The forward Clk 1 primer was 5′ATGAGACATTCAAAGAGAACTTACT 3′ and the reverse Clk 1 primer was CCGAATTCCTGCTACACGTCTACCTCCCAC. PCR conditions were 95°C for 1 min, 60°C for 1.0 min, 72°C for 1.0 min, 22–25 cycles and a 10 min soak at 72°C. Hypoxantine-guanine phosphoribosyltransferase, HPRT, was used as the control. PCR products were separated by PAGE gel electrophoresis and analyzed using the Molecular Imager (Biorad-FX).

### Oil Red O Staining

3T3-L1 cells grown in tissue culture plates were washed in PBS and 10% formalin was added for 10 min at room temperature (RT). After discarding, 2 ml fresh formalin was incubated for at least 1 hr. Cells were washed with 2 ml H_2_O (2×) and 2 ml 60% isopropanol for 5 min at RT. After drying, 1 ml of Oil Red O solution (0.35 g/100 ml isopropanol then filtered) was added, and incubated 10 min with gentle shaking. Cells were then viewed using brightfield microscopy and digital photos captured.

### Immunofluorescence Staining

3T3 L1 pre-adipocytes were grown to 80% confluence in 6-well plates on glass coverslips and plasmids indicated were transfected into cells with Lipofectamine™. After differentiation for 4 days, coverslips were washed in warm PBS and 2% formaldehyde in PBS was added to fix the cells for 15 min at 37°C. Cells were permeabilized with 10% TritonX-100 and blocked in 1% BSA with anti-goat IgG in PBS. The primary antibody was diluted 1∶100 and incubated for 1 hr in 2% BSA, 10% NP-40. After washing with PBS, the secondary antibody (anti-mouse or anti-rabbit HTC) was added in 1% BSA at room temp for 30 min. For DAPI, 1 g/ml of solution was incubated with the slides for 2 min. Slides were mounted and sealed. Images were acquired by inverted fluorescent microscopy using a Nikon Eclipse TE2000-U using a 20× objective with 0.38 µM/pixal.

## Results

### Expression of Clk1 during Adipogenesis

**Figure 1:**
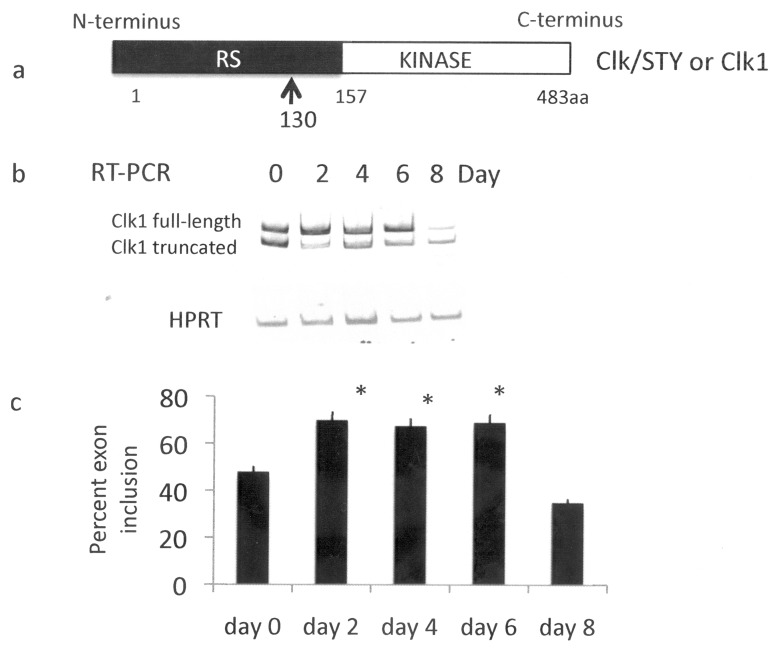
Clk1 mRNA splice variant levels in differentiating 3T3-L1 adipocytes. Pre-adipocytes were grown to confluence before initiating differentiation on day 0 with insulin, isobutylmethyxantine and methylxanthine as described in Methods. **A.** A schematic of Clk/Sty or Clk1. The alternative splice site, producing a truncated protein, is noted with an arrow. RS indicates arginine/serine rich region. **B.** Total RNA was extracted and reverse transcribed. Clk1 splice variants were amplified as described in Methods. The housekeeping gene was hypoxanthine-guanine phosphoribosyltransferase, HPRT. **C.** Data are expressed as percent exon inclusion. The asterisk indicates significance at p<0.05 by Wilcoxon test compared to day 0, N = 4.

We hypothesized that Clk1 levels were increased developmentally during adipogenesis since splicing from PKCβI to PKCβII was increased dramatically between days 4–6 of differentiation as well as expression of the two splice variants of PPARγ on day 2. Clk1 mRNA levels were measured by RT-PCR in 3T3-L1 pre-adipocytes during the course of adipogenesis. The two splice variants of Clk1 mRNA were detected. A shift in the ratio of full length to truncated form, lacking the kinase domain, occurred during adipocyte differentiation with the full-length transcript predominating on days 2–6. After terminal differentiation, the truncated form of Clk1 exceeded the full length on day 8 (Figure 1). Clk1 is alternatively spliced during differentiation of erythroleukemia cells [18]. 3T3-L1 cells showed a similar pattern in switching to the shorter truncated form after terminal differentiation.

### Inhibition of Clk Activity by TG003

**Figure 2:**
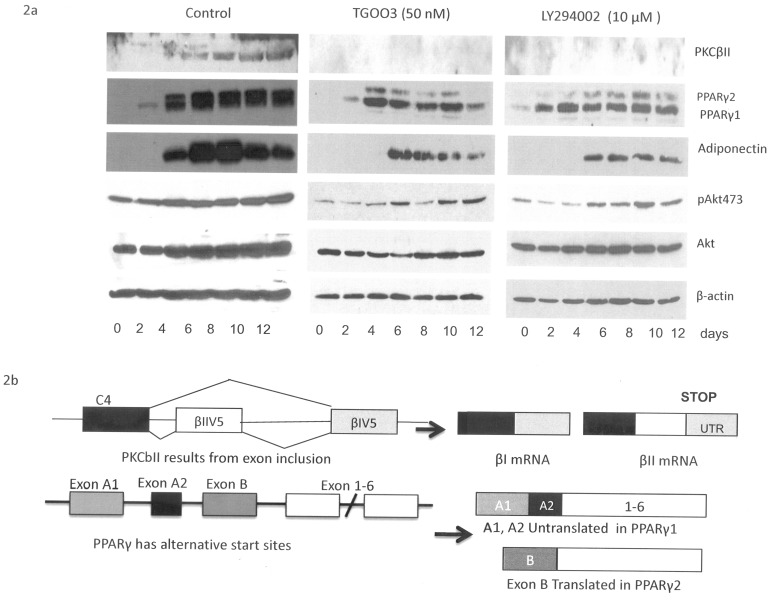
TG003 and LY294002 treatment down-regulates protein expression during 3T3-L1 adipocyte differentiation. **A.** After TG003 (50 nM) or LY294002 (10 uM) treatment for the first three days during differentiation, western blot analysis showed that protein levels of PKCβII, PPAR γ1/γ2, and adiponectin were down-regulated. Compared with control cells, phospho Akt 473 levels were down regulated by inhibitor treatment. Total Akt levels were not affected. **B.** A schematic of splice variants for PKCβ and PPARγ. Alternative splicing using βIIV5 exon inclusion produces PKCβII which renders the last exon as an untranslated region since there is a STOP codon in the βII exon, and βIIV5 exon exclusion produces PKCβI. PPAR γ has alternative start sites, Exon B is translated in PPAR γ2, Exons A1, A2 are untranslated in PPAR γ1.

We previously demonstrated that the depletion of Clk1 by siRNA or inhibition of the kinase by a general Clk inhibitor, TG003, or an upstream inhibitor of Akt activation, LY294002, blocked insulin-regulated splicing of PKCβII in muscle cells [15]. TG003 is primarily a Clk1 and Clk 4 inhibitor synthesized by Hagiwara et al [19]. It was unknown whether splicing during cellular differentiation was regulated by the PI3-kinase/Akt/Clk1 pathway. To study this, we exposed 3T3-L1 pre-adipocytes to 50 nM TG003, an inhibitor of Clk 1 (EC50 20 nM) and Clk 4 (EC50 15 nM), and LY294002 (10 µM), an inhibitor of phosphatidyinositol 3 (PI3)-kinase, on days 1–3 of differentiation. TG003 blocked PKCβII expression during differentiation (Figure 2a). PKCβII pre-mRNA splicing, shown in Figure 2b, was previously shown to be responsible for its expression [20]. This indicated that Clk kinase activity was required for PKCβII splicing in adiogenesis. LY294002 treatment of pre-adipocytes also blocked PKCβII expression, and confirmed the activation of the PI3Kinase/Akt/Clk1 pathway as shown previously in L6 muscle cells for adipogenesis [15].

There were also differences in the expression of other alternatively spliced proteins with TG003 treatment. The alternative splicing of PPARγ is due to the use of alternative promoters as depicted in Figure 2b. PPARγ2 variant includes a translated exon and the protein migrates above PPARγ1 on western blots. Figure 2b illustrates the scheme of splice variants for PKCβ and PPARγ pre-mRNA. As shown in Figure 2a, TG003 treatment altered the expression of PPARγ1 (lower band) and PPARγ2 (upper band), and delayed the expression of adiponectin. Another inhibitor of Clk1 and 4, DRB, known to block Clk1 and 4 isoforms [21], acted in a similar manner to TG003 (data not shown). LY294002, however, primarily blocked PPARγ2 expression. These results suggested that mechanisms were initiated early in the differentiation program, prior to PPARγ expression, to regulate splicing and expression of adipogenic proteins via Akt activation of Clk1 kinase.

### Effects of Clk1 siRNA Treatment of Cells

**Figure 3:**
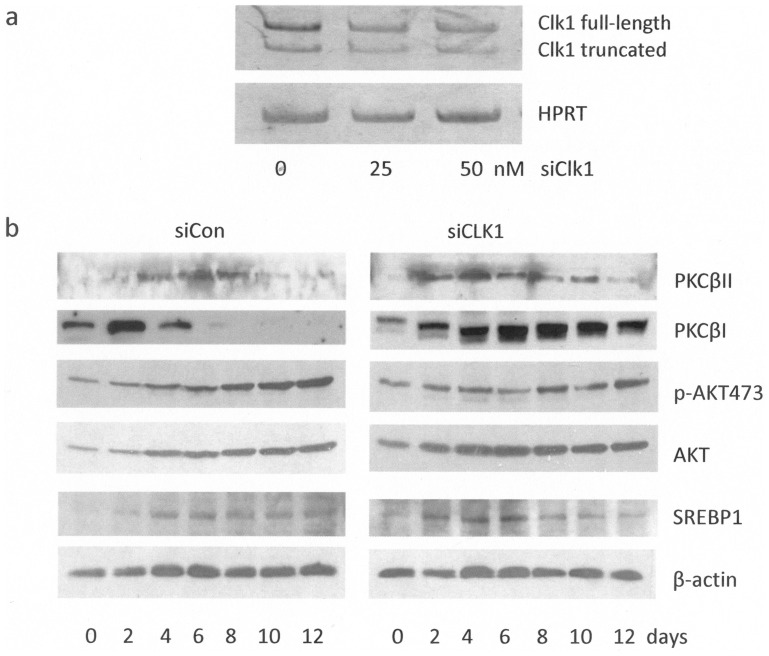
Clk1 mRNA levels in siRNA treated 3T3-L1 cells. **A.** The Clk1 siRNA oligos were tranfected into 3T3-L1 pre-adipocytes and total RNA was extracted and reverse transcribed after 4 days of differentiation. The levels of full length Clk1 were decreased 50% by siRNA at 25 (nM) and 50 (nM). **B.** Clk1 depletion by siRNA alters protein expression during 3T3-L1 adipocytes during differentiation. Protein lysates from cells treated with SiRNA to Clk1 were differentiated for 12 days as described in Figure 1, and subjected to SDS-PAGE and western blot analysis with antibodies as indicated in the right-hand legend. The experiment was repeated twice to ensure reproducible results.

Since TG003 was not a specific inhibitor for Clk1, Clk1 siRNA was used to knock down the isoform that was shown to be upregulated with differentiation. Clk1 was focused on as it was known to be important for SRp40 phosphorylation and PKCβ splicing [15]. Clk1 siRNA depleted cells of 75% of the target compared to control siRNA treated cells as determined by Western blot (Figure 3a). siRNA to Clk1 had no effect on Akt levels but diminished pAkt473 in differentiating adipocytes (Figure 3b). Elevated PKCβII levels were detected as early as day 0 and PKCβI levels remained elevated throughout differentiation. The expression of SREBP-1, an upstream transcriptional activator of adipogenesis, was not significantly altered by siCLK1. The reduction of cellular Clk1 levels had a different effect on splicing of PKCβII than did the inhibition of Clk kinase by TG003 or LY294002. The depletion of Clk1 levels also served to reduce the cells of an important Akt substrate that resulted in dysregulated or increased PKCβII & PKCβI levels.

### Mutation of Akt Sites in Clk1 to Alanine

Clk1 kinases are conserved throughout eukaryote evolution from Saccharomyces cerevisiae (KNS1), Arabidopsis thaliana (AFC1-3), and Drosophila melanogaster (DOA), mouse (Clk/Sty) to human (Clk1,2,3,4) [22]. To determine the effect of Akt phosphorylation sites in Clk1 (Clk/STY), site directed mutagenesis of all three Akt sites was initially performed. These sites are not located in the C-terminal kinase domain but are in the N-terminal domain lobe that is described as containing an RS domain with multiple arginine-serine dipeptides as shown in Figure 1. Akt phosphorylation sites were identified using PhosphoMotif Finder in the Human Protein Reference Database (hprd.org). The N-terminal lobe of the enzyme consists of three β strands (β1-β3) followed by an α helix (αc) and two further β strands β 4 and β 5). The C-terminal domain exhibits features defining the Clk family including the highly conserved signature motif of the EHLAMMERILG, from which the LAMMER-kinases is named [23]. This domain contains the ATP binding site and the active site lysine (Lys191). The activation loop of Clk1 is defined by the electron density and adopts the conformation of active kinases without phosphorylation [23].

**Figure 4:**
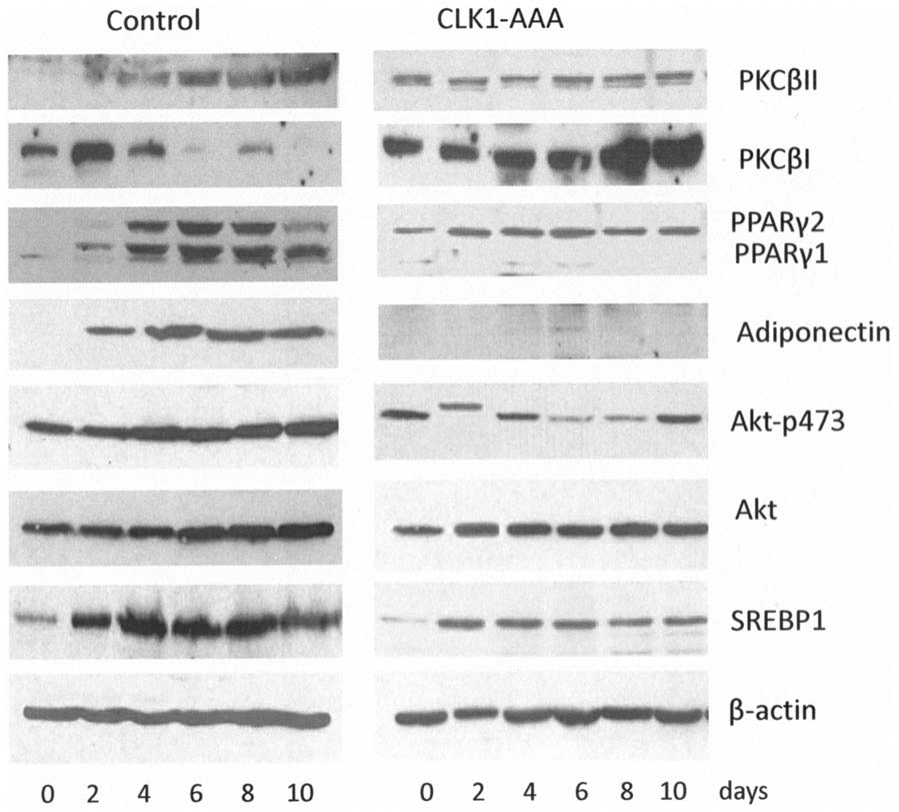
Clk1 triple mutant-AAA, alters PKCβ and PPARγ splice variants, adiponectin, Akt, and SREBP1 expression in 3T3-L1 cells during differentiation. Cells were grown and differentiation initiated as described in Figure 1 with the exception that Clk1-AAA cDNA or empty plasmids were transfected into cells as described in Methods. Protein lysates were collected on days 0–10 and separated on SDS-PAGE, transferred to membranes, block, and exposed to antibodies indicated on the right hand legend. Shown are representative gels from experiments repeated at least twice to ensure reproducible data.

The role of the n-terminal lobe in substrate specificity is only speculated, however, as a kinase inactive mutant, Clk1 colocalizes with other splicing factors to nuclear speckles causing them to dissolve. Here, we mutated only the Akt sites in Clk1 predicted to be specific for recognizing SR proteins, while leaving the kinase domain active. In this experiment, we looked at the ability of Clk1-AAA (S36, T122, and S139 were all mutated to A) mutants to regulate PKCβ and PPARγ splicing and adiponectin expression as a measure of adipogenesis. When Clk1-AAA was transfected into 3T3-L1 preadipocytes, PKCβII levels were elevated on day 0 and throughout differentiation (Figure 4). PKCβI levels were also sustained throughout the differentiation process. PPARγ1 levels were depleted compared to the control. Akt expression and SREBP 1 expression were unaffected by the triple mutant Clk1-AAA. This indicated Clk action was downstream of SREBP1 transcription, however, pAkt473 levels were diminished in early adipogenesis suggesting a feedback on its activation.

### Single Mutations of Akt Phosphorylation Sites in Clk1

**Figure 5:**
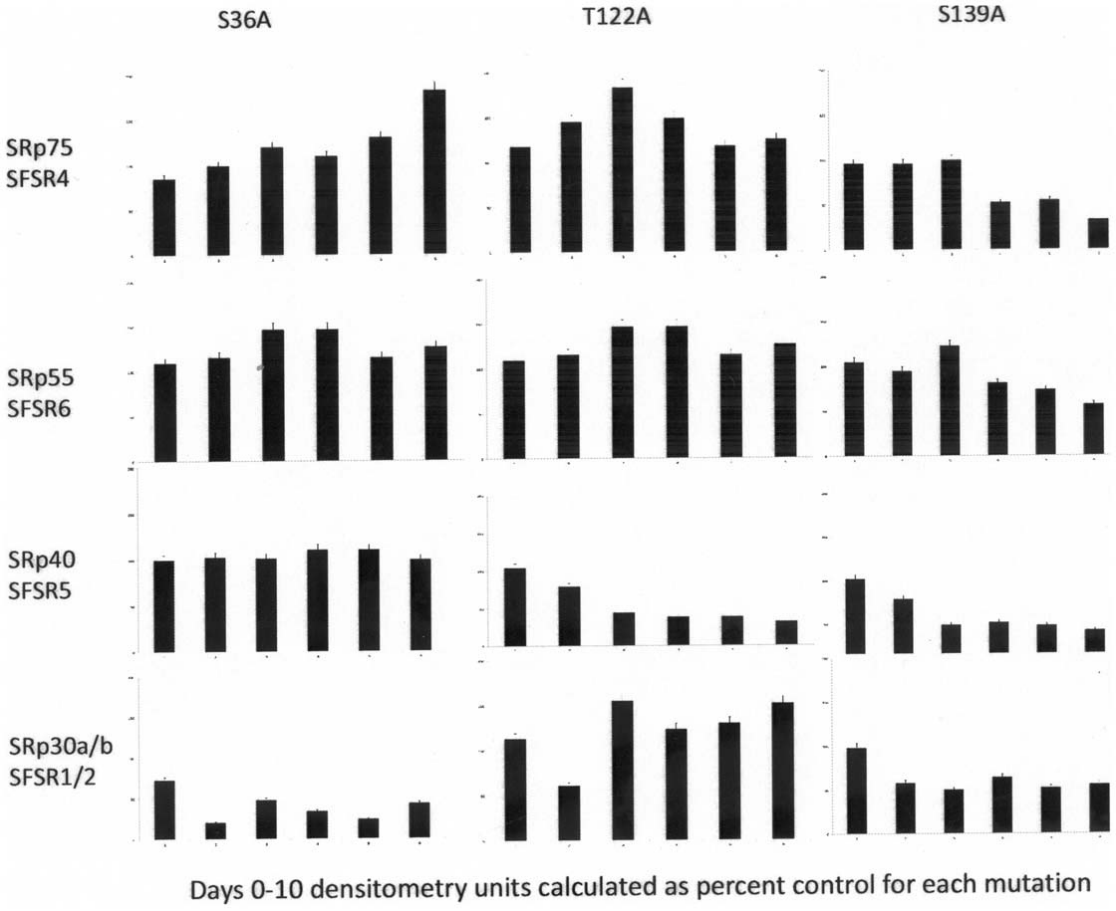
Single mutant sites on Clk1 cDNA alter phosphorylation of SR proteins. Plasmids of single Akt site mutants of Clk1 were transfected into 3T3-L1 cells. Clk1 substrate phosphorylation levels were probed using a monoclonal antibody recognizing the Clk phosphorylation motif, mAb104, during differentiation. Protein lysates were subjected to western blot. Densitometric scans of each SR protein band corresponding to SRp75 (SFSR4), SRp55 (SFSR6), SRp40 (SFSR5) and SRp30a/b (SFSR1/2) were compared to the control blot and were plotted as percent control for each mutation. For S36A, scans indicated SFSR1/2 was less phosphorylated than other SR proteins of interest. For T122A, scans indicated SFSR5 was the least phosphorylated of the SR proteins. For S139A, all proteins demonstrated some loss of phosphorylation compared to the control blot. The experiment was repeated twice to ensure reproducible results.

Since the triple mutation of Akt sites on Clk1 (Clk1-AAA) had multiple effects on splicing and protein expression, individual site mutations of Akt phosphorylation motifs were made and the single mutant Clk1 plasmids were transfected into 3T3-L1 cells and cell lysates were isolated on days 0–10 during differentiation for Western blots of Clk1 phosphorylation activity using and antibody that recognizes the phospho-Clk substrate motif, mAb104 [15]. This antibody assay is preferable to a conventional kinase assay since activity cannot be confused with other interacting kinases such as Akt or PKC that can modify Clk activity. Phosphorylated SR proteins were identified by their molecular weights and by immunoprecipitation and reference immunoblot [15] Mutation of individual Akt phosphorylation sites in Clk1 demonstrated that each mutation resulted in diminished phosphorylation of one or more SR proteins which were analyzed densitometrically and compared to the control containing non-mutated Clk1 (Figure 5). Site mutation S36A resulted in reduction of SRp30a/b (also known as SFSR1 and SFSR2) phosphorylation. SRp30a/b are two closely related SR proteins with regard to their migration as a doublet on western blot and they are also referred to using new and old nomenclature as SFRS1/SRp30a (ASF/SF2) and SFRS2/SRp30b (SC35). The phosphorylation of the other three SR proteins remained intact as shown in the scans. Site mutation T122A resulted in reduction of SRp40 (also known as SFRS5 or HRS) phosphorylation relative to SRp55, SRp75, and SRp30a/b phosphorylation when compared to control. Site mutation S139A resulted in reduction of SRp75 (SFRS4), SRp55 (SFRS6) and SRp40 (SFRS5) as well as SFRS1 and SFRS2 phosphorylation by Clk1-S139A. Each site mutation reduced specific SR protein phosphorylations with S139A having the greatest effect on all SR proteins shown here. The apparent increase in phosphorylation noted on days 4–6 is attributed to increased protein levels of some SR proteins which is known to occur.

### Akt Site Mutations on Clk Dysregulate PKCβII

**Figure 6:**
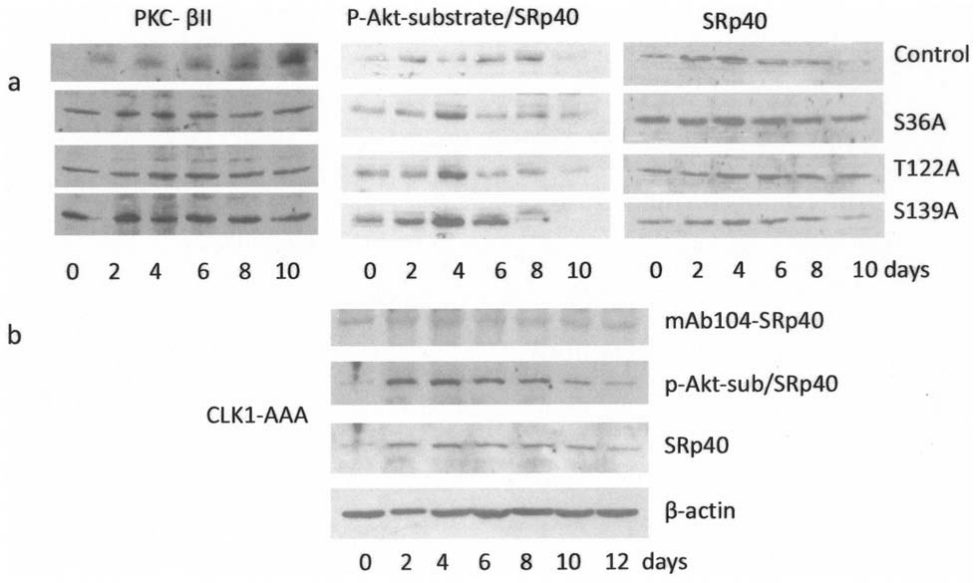
Single Akt-site mutations on Clk1 dysregulate PKCβII and increase Akt phosphorylation of SRp40. **A.** 3T3-L1 cells were transfected with single mutant Clk1 constructs as indicated and PKCβII levels, phosphoAkt-substrate for SRp40 and SRp40 levels were probed as indicated in cell lysates collected from cells exposed to differentiation conditions. **B.** 3T3-L1 pre-adipocytes were transfected with Clk1-AAA mutant and probed for phosphorylation of SRp40 by Clk1 using mAb104, phosphoAkt substrate antibody to detect Akt phosphorylation of SRp40, and SRp40 antibody in cell lysates from cells exposed to differentiation conditions. Transfections were performed on multiple occasions to ensure reproducible results.

**Figure 7:**
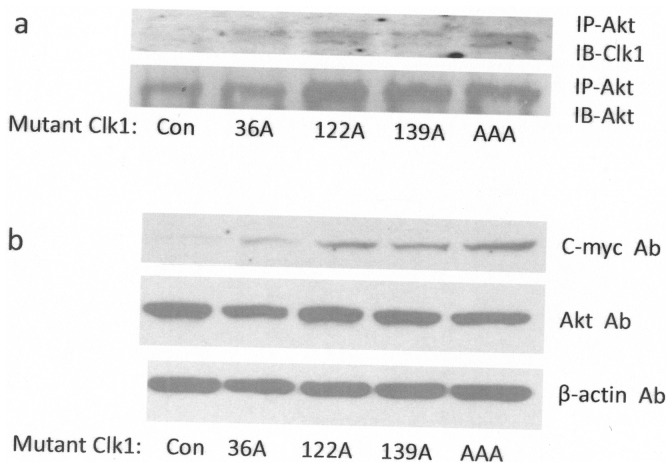
Expression levels of mutant Clk1 and immunoprecipitation analysis of interactions of mutant Clk1 with Akt. **A.** 3T3-L1 cells were transfected with control (empty plasmid) and phospho-Akt site mutant Clk1 as indicated. Proteins were immunoprecipitated by Akt antibody, blot were then probed with either Clk1 or Akt antibody. **B.** Transient expression levels of mutant Clk1 levels were detected on blots by C-myc antibody, endogenous Akt was also detected, and β-actin was used as a loading control.

We previously demonstrated the role of Akt2 and Clk/STY in regulation PKCβ alternative splicing via SRp40 [15]. Akt2 can phosphorylate SRp40 directly and indirectly through Clk. Hence, we studied the effects of mutating Akt sites on Clk, phosphorylation of SRp40 and PKCβII levels during 3T3-L1 cell differentiation to mature adipocytes. High levels of PKCβII expression occurred early during differentiation when mutant Clk1 cDNAs were transfected into pre-adipocytes with the highest level of expression occurring in Clk1-S139A mutant cells (Figure 6). Akt phosphorylation of SRp40 was detected using the phospho-Akt substrate antibody while phosphorylation of SRp40 by Clk1 was detected using mAb104, the phospho-Clk substrate antibody [15]. Clk1-S139A increased Akt phosphorylation of SRp40 more than any other mutation. The Clk1-AAA (triple) mutant also increased Akt phosphorylation of SRp40 (Figure 6b). Mutant Clks were still able to associate with Akt as demonstrated by the co-immunoprecipitation of Clk1-S36A, Clk1-T122A, Clk1-S139A, and Clk1-AAA with Akt (Figure 7a). Expression of mutant Clks is shown via c-myc expression (Figure 7b). These results indicated that mutating individual Akt phosphorylation sites on Clk1 reduced phosphorylation of some SR proteins. Counter to the decreased Clk phosphorylation of SRp40, there was increased Akt phosphorylation of SRp40 with mutant T122A and S139A, and an increased early expression of PKCβII was noted on days 0–4 in pre-adipocytes.

### Akt Site Mutations on Clk1 Alter Adipogenesis

**Figure 8:**
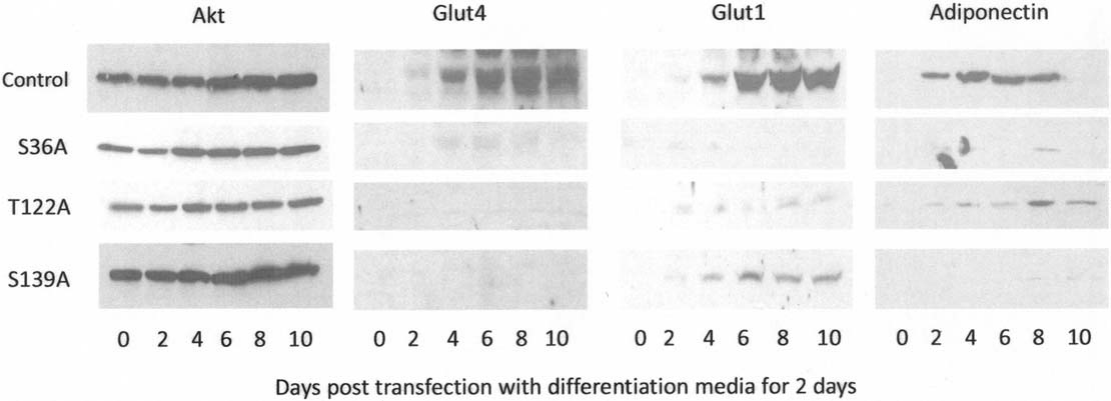
Clk1 mutants alter adipogenesis. Plasmids of Clk1 with single mutations of Akt phosphorylation site were transfected into 3T3-L1 cells and protein lysates collected after treating cells with differentiation conditions were blotted and probed for Glut4, Glut1 and adiponectin. The experiment was performed on two occasions with similar results.

Expression of major adipogenic proteins was probed by western blot in cell lysates obtained at days 0–10 during differentiation following transfection of the single Clk1 mutants. The adipogenesis program was blocked in cells transfected with the single Clk1 mutants prior to differentiation. As with Clk1-AAA, when all three Clk sites were mutated in a single construct, and adiponectin expression was blocked (as shown in Figure 3), single mutations resulted in blocking adiponectin, Glut4 and Glut1 expression. With the exception of Clk1-T122A, where low adiponectin expression was noted later in differentiation, and Clk1-S39A where low Glut1 expression was noted, all cells failed to undergo adipogenesis as indicated by expression of these proteins (Figure 8). This suggested that specific SR proteins and splicing events orchestrated initial differentiation of 3T3-L1 cells.

### Clk1 Mutants Fail to Form Lipid Droplets

**Figure 9:**
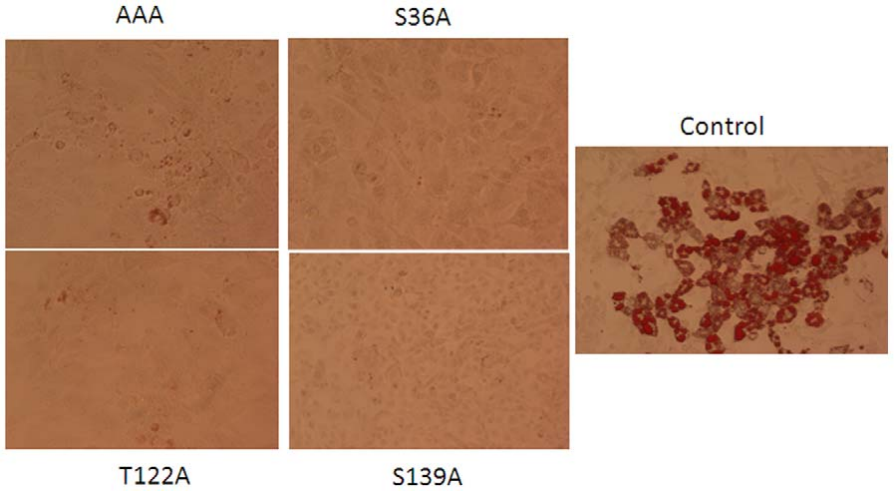
Oil Red O staining of 3T3-L1 cells after day 6 of transfection with Clk1 mutants. 3T3-L1 cells were grown on 35 mm dishes and transfected with Clk1 mutants as labeled. Following 6 days of the differentiation procedure for adipogenesis, cells were stained with Oil Red O as described in Methods and brightfield microscopy revealed the extent of lipid accumulation compared to control (plasmid only) transfected cells.

Figure 9 depicts 3T3-L1 pre-adipocytes transfected with the Clk1 mutants after day 6 of differentiation. Oil Red O staining shows that each mutation blocked the accumulation of lipids indicating a block in adipocyte differentiation potential.

### Clk1-AAA Mutant Excludes SRp40 from the Nucleus during Adipogenesis

**Figure 10:**
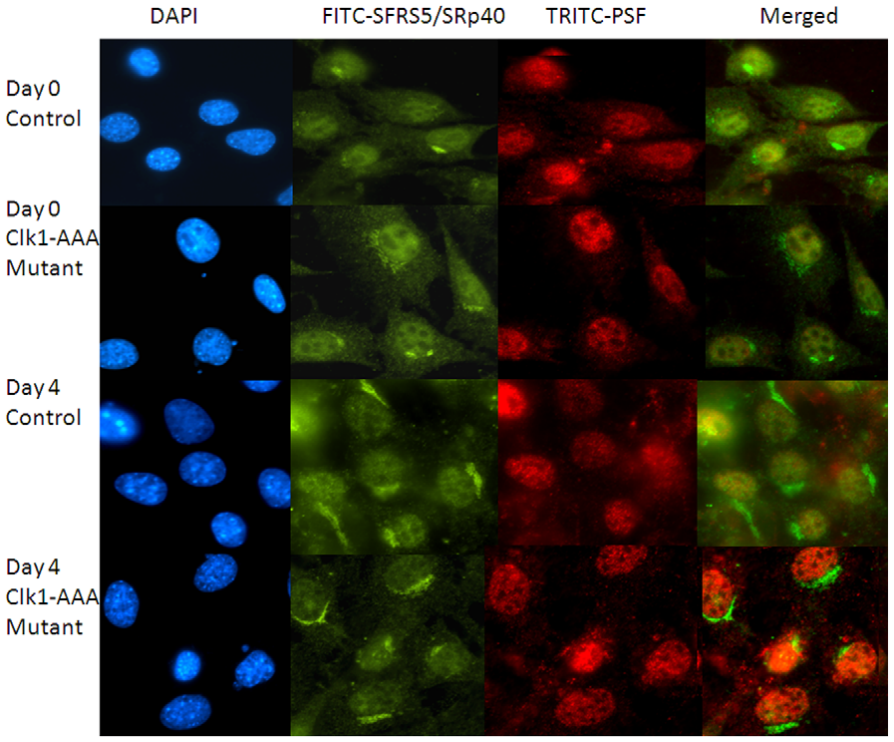
Clk1-AAA mutant excludes SFRS5 (SRp40) from nuclear localization following differentiation conditions. 3T3-L1 cells were transfected with Clk1-AAA cDNA or control plasmid and then exposed to differentiation conditions for 4 days prior to fixation and staining with DAPI or conjugated SFSR5 (or SRp40) or PSF antibodies for fluorescence microscopy. Conditions of image acquisition are described in Methods. The experiment was performed twice to ensure reproducible results.

We hypothesized that Clk phosphorylation of SR proteins was necessary for their nuclear localization during splicing and that the Clk1-AAA mutant would result in excluding splicing factors from nuclear paraspeckles. To examine this, 3T3-L1 pre-adipocytes were transfected with Clk1-AAA cDNA and differentiated through day 4. Cells were examined on day 0 (24 hr post-nucleofection) and day 4 after 2 days in induction media. We visualized SRp40, as in our lab it is known to regulate PKCβII alternative splicing. As a control, cells were stained with a nuclear stain, DAPI, FITC conjugated SRp40 (SFRS5), and TRITC conjugated PSF, a PTB-associated splicing factor, and a mouse and human paraspeckle-associated protein [24]. When FITC-SRp40 and TRITC-PSF frames were merged, the images indicated that on day 0, both SR040 and PSF colocalized to nuclear perispeckles in control and Clk1-AAA mutant transfected cells (Figure 10). However, by day 4, SRp40 was overexpressed in both control and the Clk1-AAA mutant cells, and it was largely perinuclear. In the Clk1-AAA mutant transfected cells, however, SRp40 was excluded from the nucleus. This is a crucial day for splicing regulation. In control cells, both PSF and SRp40 colocalized to discrete speckles within the nucleus on day 4. Thus, mutation of Akt sites on Clk prevented SRp40 from undergoing colocalization with PSF to nuclear speckles during differentiation.

## Discussion

The functional role of Clk kinase in alternative splicing is mediated by its substrates, serine-arginine rich “SR” splicing factors. Clk1 (also known as Clk/STY in mouse) is regulated by Akt phosphorylation in response to insulin [15]. Here, we examined levels of Clk1 and its truncated splice variant during the course of murine 3T3-L1 pre-adipocyte differentiation. Clk1 regulates its own splicing leading to two transcripts encoding the full-length catalytically active kinase and the truncated form lacking a kinase domain [25], [26]. The long or active form of Clk1 prevailed except for Days 0 and 8, when cells were not undergoing differentiation. Erythroleukemia cell differentiation showed a bias toward the skipped or truncated splice variant post-commitment [18], and this was noted during adipogenesis in 3T3-L1 cells. Full length Clk1 expression was up-regulated during hormone induced 3T3-L1 cell differentiation, however, the ratio of the two transcripts changed with differentiation.

Clk1 kinase phosphorylates and colocalizes with nuclear splicing factors [27], [28]. Splicing factors are identified by their phosphorylation state for nuclear compartmentalization. Presently, the Clk inhibitor, TG003, was used to block activity during adipogenesis. In addition to blocking PKCβII splicing, we also found that the expression of PPARγ1&2 and adiponectin was blocked. TG003 inhibits both Clk1 and 4 isoforms at the concentration used here. Hence, Clk1 and 4 activities were crucial for expression of adipogenic proteins, i.e., PPARγ and PKCβ, and other alternatively spliced proteins that promote cellular differentiation [24].

Clk kinase activity is essential for phosphorylation of the splicing factors that recognize *cis-*elements within exons [29], [30], and also participate in co-transcriptional splicing. The N-terminal domain of Clk contains a serine/arginine-rich domain that recognizes SR proteins [30]. Both the full length and truncated Clk recognize SR proteins. The finding that mAb104 recognizes a phosphoepitope in SR proteins paved the way for elucidating how Clk might be regulated [15], [31], [32], [33]. Our work showed that mAb104 recognized the phospho-Clk motif in proteins and that Akt regulates Clk1. Here, two approaches depleted Akt phosphorylation sites in Clk to determine how it could alter SR protein activity in splicing during differentiation.

First, siRNA was used to deplete Clk1 in pre-adipocytes. This depleted cells of both Akt substrate motifs and the kinase activity of one Clk isoform. It resulted in the early expression of PKCβII and continued expression of PKCβI beginning on day 0 of differentiation. Hence, a dysregulation of splicing was observed in contrast to the tight regulation governing the timed switch from one splice variant to the other between days 4–6. There was also depletion of PPARγ1 and a reduction in PPARγ2 expression, and this was also different from the inhibition observed with TG003, an inhibitor of both Clk1 and 4 kinases. Thus, without Clk1, cells failed to produce a transcription factor known to induce adipogenesis, PPARγ. It was also noted that mAb104, a phospho-Clk substrate antibody, no longer recognized a heavily phosphorylated SRp40 band despite no changes in its protein expression. However, in the absence of Clk1, SRp40 became a better substrate for Akt phosphorylation and this suggests the inability of SR proteins to co-localize to the nucleus for further Clk1 phosphorylation once they have been phosphorylated by Akt [31].

The mutation of Clk1 on all three Akt phosphorylation sites leaving its kinase domain intact, produced similar results to siRNA. PKCβII was heavily expressed early on and PPARγ1 expression was depleted. Overexpression of the triple Clk1 mutant had a similar effect to the depletion of Clk1 using siRNA for Clk1. Without available Akt sites in Clk1, SR proteins were not properly phosphorylated resulting in a disruption of splicing. It was also noted here that SRp40 did not colocalize to nuclear speckles with PSF. The dysregulation of PKCβII is likely due to improper SRp40 phosphorylation by Clk1 and the inability of the SR protein to interact with RNA PolII during transcription. During adipogenesis, the splicing of PKCβII is regulated developmentally via co-transcription [17]. ChIP analysis of the PKCβ promoter revealed that SRp40 and PU.1 associated with promoter elements in 3T3-L1 pre-adipocytes [17]. The selectivity of Clk1 for splicing of PPARγ1 vs γ2 will require more studies, but suggest for the first time that different Clk kinase isoforms regulate PPARγ1 & 2 expression in early adipogenesis. Clk4 appeared to be responsible for PPARγ2 alternate promoter transcription where Clk1 siRNA was related to depletion of PPARγ1 expression based on studies using TG003 to inhibit both kinase activities. Blocking of PPARγ1/2 or the overexpression of PKCβII resulted in blocking adiponection expression.

To further determine the role of each Akt site in Clk1 recognition of SR proteins, single Akt sites were mutated and constructs transfected into 3T3-L1 pre-adipocytes. Single site mutations resulted in different developmental patterns of SR protein phosphorylation. In control cells, identification of five predominant phosphorylated SR proteins is possible given their molecular weights and mobilities on SDS-PAGE. When Serine36 was mutated to alanine, SRp30a and b protein phosphorylation was reduced. This protein doublet represents ASF/SF2 (SFSR1) and SC35 (SFSR2) proteins, SR proteins known to function in constitutive splicing and alternative splicing. When Threonine 122 was mutated, the phosphorylation of SRp40 (SFSR5) was disrupted. The Clk phosphorylation states of all five proteins (including SRp55 (SFSR6) and SRp75 (SFSR4)) were significantly blocked when Serine 139 was mutated. Each mutation increased PKCβII expression and increased the phosphorylation of SRp40 by Akt as was also demonstrated with Clk siRNA. None of the mutations, however, interfered with the affinity of Akt for Clk1 or altered SRp40 levels or any other SR protein levels. The block of adipogenesis by single mutations in Clk1 was documented by lack of Glut4, Glut 1 and adiponectin expression as well as failure of the cells to develop Oil Red O staining lipid droplets.

Since phosphorylation of SR proteins by Clk is thought necessary for their nuclear roles, Clk1-AAA, was used to demonstrate this. Immunfluorescence microscopy showed that another nuclear paraspeckle protein, PSF, no longer co-localized with SRp40 when mutant Clk1-AAA was expressed during adipogenesis. The SRp40 remained perinuclear unlike the SRp40 in control cells which was detected in nuclear as well as in distinct perinuclear areas.

The finding that Clk1 is involved in adipogenesis is not surprising as the kinase was discovered while searching for tyrosine kinases involved in differentiation programs [34]. Its role in differentiation is through modulation of gene expression [18]. The major substrates of Clk kinases are SR splicing proteins [30]. We hypothesized that the phosphorylation of multiple SR proteins was involved with the expression of alternatively spliced transcripts that shepherd the preadipocyte into its fully differentiated state. At the core of Clk’s activity is its recognition of SR proteins which was shown to be regulated by Akt phosphorylation. Akt1 is important in adipogenesis [35], [36]. However, its role has been primarily linked to FOXO transcriptional regulation. The finding that Akt regulation of Clk1 regulates adipogenesis extends its role in cellular differentiation. Regulation of SR protein phosphorylation is not well understood even though splicing factors are essential components of the splicing machinery that exert their function through phosphorylation/dephosphorylation [37]. The RS domain contains multiple repeats that can be phosphorylated by both Akt and Clk [29], [38]. Clk also contains these RS domains. This report shows that Akt phosphorylation of Clk regulates the subcellular localization of SR splicing factors and provides a means of further directing their localization during differentiation.

The orchestration of Akt and Clk to regulate splicing emerges here when the Akt sites on Clk1 are mutated and the expression of key adipogenic proteins are disrupted including PPARγ, Glut4, Glut1, and adiponectin. This pathway is likely to be important not only is adipogenesis, but in other lineage specific commitment pathways. Mutation of similar Akt sites in Clk2 resulted in cells that were no longer resistant to survival following ionizing radiation [39]. The splice variants of PPARγ may contribute to the co-transcriptional splicing of proteins since PPARγ, SRp40 and PGC1α were shown to interact to regulate splicing [40]. The recognition of other SFSR or SR proteins by similar Akt sites in Clk2, 3, and 4 will likely explain the plasticity and ability of cells to regulate splicing events during differentiation.
